# Association mapping and identification of candidate genes for callus induction and regeneration using sorghum mature seeds

**DOI:** 10.3389/fpls.2025.1430141

**Published:** 2025-04-24

**Authors:** Jingyi Xu, Lihua Wang, Yuan Liang, Qi Shen, Wenmiao Tu, Zhengxiao Cheng, Lu Hu, Yi-Hong Wang, Jieqin Li

**Affiliations:** ^1^ College of Agriculture, Anhui Science and Technology University, Fengyang, Anhui, China; ^2^ International Joint Research Center of Forage Bio-Breeding in Anhui Province, Chuzhou, China; ^3^ Department of Biology, University of Louisiana at Lafayette, Lafayette, LA, United States

**Keywords:** tissue culture, regeneration, GWAS, HDG5, WIND1

## Abstract

**Introduction:**

A whole plant can be regenerated through tissue culture from an embryogenic callus in a process referred to as plant regeneration. Regeneration ability of embryogenic callus is a quantitative trait and the main limiting factor for genetic studies in sorghum.

**Methods:**

We evaluated 236 sorghum mini core varieties for callus induction rate, embryogenic callus rate, callus browning rate and differentiation rate and performed a multi-locus genome-wide association study (GWAS) of the four traits with 6,094,317 SNPs.

**Results:**

We found five mini core varieties most amenable to tissue culture manipulations: IS5667, IS24503, IS8348, IS4698, and IS5295.Furthermore, we mapped 34 quantitative trait loci (QTLs) to the four traits and identified 47 candidate genes. Previous studies provided evidence for the orthologs of 14 of these genes for their role in cellular function and embryogenesis and that the ortholog of WIND1 (WOUND INDUCED DEDIFFERENTIATION 1) identified in this study promotes callus formation and increases de novo shoot regeneration.

**Conclusion:**

These candidate genes will help to further understand the genetic basis of plant embryonic callus regeneration.

## 1 Introduction

Sorghum [*Sorghum bicolor* (L.) Moench] is a highly valuable and adaptable crop, playing key roles in food, feed, biofuels, and even traditional alcoholic beverages like baijiu in China (Zheng et al., 2023). The challenges in sorghum’s tissue culture and genetic transformation, particularly the browning of calli, highlight the complexity of working with this plant for biotechnological advancements (Zheng et al., 2023; Silva et al., 2022). Factors like genotype-dependent phenolic production contribute to the difficulty, making it a recalcitrant species for genetic modification (Flinn et al., 2020). This presents an interesting area of study for overcoming tissue culture barriers to improve sorghum’s seed quality, which is crucial for its various uses, especially in baijiu production. Sorghum recalcitrance to tissue culture has been demonstrated by several studies. An evaluation of five sorghum varieties has showed a plantlet regeneration frequency of 0-2.8% from immature embryo (Ahmed et al., 2021). Another study evaluated 10 sorghum varieties and attained embryogenic callus rate of 13.4-47.5% across media also from immature embryo (Assem et al., 2014). Culturing immature embryos from Keller, Kansas, Atlas and Fortuna found callus induction rate of 1-84% in their best media (Espinoza-Sánchez et al., 2018). An embryogenic callus rate of 10-89% was reported for immature embryos from 32 sorghum varieties, depending on varieties and media (Raghuwanshi and Birch, 2010).

Although immature embryos were frequently used, their isolation is labor-intensive and transformation efficiency using immature embryos harvested in winter can be reduced by 1/3 (Do et al., 2016). In contrast, mature seeds represent an attractive alternative because they are independent of seasonal effects and convenient to access (Wang et al., 2021a). Using mature seeds from sorghum varieties GK Emese, GK Zsófia, and Róna 1, a plantlet regeneration rate of 6.7-24.4% was recorded (Chege et al., 2019). Although callus induction rate from mature seeds can be very high (10-87%), none of the calli regenerated (Murty et al., 1990). In a screening of 250 sorghum varieties, although mature seeds from 245 varieties produced calli, only calli from 47 varieties regenerated shoot and root (Li et al., 2017). We have previously cultured mature seeds from 120 mini core landrace varieties and found that 25 (21%) of the varieties could be induced to produce callus and 23 of the 25 showed low callus browning rate (Li et al., 2017).

Previous studies have mapped callus induction (Tuskan et al., 2018)and callus and shoot regeneration (Nagle et al., 2024) in poplar, callus and shoot regeneration in barley (Tyagi et al., 2010), callus formation in rice (Zhang et al., 2019), and callus induction rate and regeneration in maize (Ma et al., 2018) and identified possible genes underlying embryogenesis. These are important as they may facilitate breeding for genotypes more amenable to *in vitro* plant regeneration and genetic transformation, in addition to elucidating genetic control of induced embryogenesis. We evaluated 236 sorghum landrace varieties of the globally sampled mini core collection (Upadhyaya et al., 2009) for callus induction, callus browning, embryogenic callus induction and callus differentiation from mature seeds. The sorghum mini core collection has been previously used to clone a gene pleiotropic for sorghum biomass and sugar yield (Upadhyaya et al., 2022) and to map sorghum panicle architecture (Wang et al., 2021b) and sorghum plant color (Wang et al., 2024). To our knowledge, there have been no reports on mapping tissue-culture-based genetic transformation related traits in sorghum.

In this study, we have two objectives. First, we want to find highly competent sorghum varieties for genetic transformation from a global germplasm collection. Second, we want to understand the genetic mechanism behind embryogenesis even though the process can be affected by hormones. We performed a GWAS on the four traits with 6,094,317 SNP markers using the GWAS method as previously described (Li et al., 2018) and mapped eight callus induction loci, one locus each for callus browning and embryogenic callus induction, and 24 differentiation loci, identified 47 candidate genes from the loci and provided evidence that orthologs for 14 of the genes play roles in cellular function and embryogenesis. To our knowledge, this is the first such report on mapping tissue-culture-based genetic transformation related traits in sorghum.

## 2 Materials and methods

### 2.1 Plant material

A total of 236 sorghum mini core varieties (**Supplementary Table S1**) were evaluated for the traits described below Each variety was planted in a row with 10 plants in each row. The row spacing was 50 cm and the plant spacing was 20 cm. In order to avoid cross-pollination, all panicles were wrapped in paper bags before flowering. After seeds grew mature, they were harvested for tissue culture.

### 2.2 Tissue culture method

We selected 90 mature seeds from each of the 236 varieties for three replicates. The seeds were washed with 70% alcohol for 10 minutes, rinsed with sterile water once, then washed with 0.1% HgCl_2_ solution and 2-3 drops of Tween 20^®^ for 15 minutes, rinsed with sterile water for 5-6 times in an ultra-clean bench until no foam was observed, and placed in a mature seed induction medium (**Supplementary Table S2**) for 10-14 days. The sample was incubated at 28°C with 70% humidity(PGX-250, Shanghai, China) without light, and then the varieties that could produce callus were selected. After subculture, callus was transferred to regeneration medium with 16h light and 8h dark at 28°C.The embryo surface of sorghum seeds was oriented downward, with 30 seeds placed per Petri dish. If callus tissue developed, the seed and bud parts were excised and transferred to a subculture medium. After 7-10 days, the medium was replaced with a differentiation medium (**Supplementary Table S2**). The seeds and embryonic callus were then cut and subcultured. Within seven days of subculture, the ability to generate embryonic tissue and the browning rate were recorded (Li et al., 2018).

### 2.3 Phenotypic measurement

We evaluated the callus induction rate of mature seeds from 236 sorghum varieties. Varieties that produced translucent callus were considered capable of generating inducible embryonic calli. During the tissue culture of sorghum mature seeds, we found that some varieties lacked callus induction ability, resulting in water-soaked callus that could not form embryonic callus. These varieties also exhibited low callus differentiation rates and browning. The callus most suitable for sorghum genetic transformation was a granular, compact, yellowish, embryogenic callus. Therefore, washed seeds were placed on the induction medium (**Supplementary Table S2**), and those that produced translucent callus were considered inducible material. After 7 days of subculture, a relatively hard, pale yellow, granular callus formed, which was identified as embryogenic callus. During this period, although embryogenic callus could grow, brown phenolic substances accumulated around the callus, leading to browning (Flinn et al., 2020). Callus that could differentiate on the differentiation medium (**Supplementary Table S2**) was considered differentiated materially. The induction rate was calculated as the number of induced calli divided by 90. The browning rate was the proportion of browning calli relative to the total number of induced calli. The embryogenic callus rate was the proportion of induced calli that developed into pale yellow granular callus, relative to the total number of calli. The differentiation rate was the proportion of regenerated plants relative to the total number of calli.

### 2.4 Data processing

The steps of DNA extraction are as follows: Firstly, the fresh leaves were grinded with liquid nitrogen, and the DNA lysis solution(**Supplementary Table S3**)was added. The cytoplasm was broken and dissolved in 65 °C water bath for 10 minutes, so that the DNA was released into the solution. Centrifugation at 12000 rpm for 4 minutes to absorb the supernatant, and then the supernatant was added to the magnetic beads, DNA binding solution(**Supplementary Table S3**) and isopropanol. After the oscillation was uniform, it was placed on the magnetic frame for 2 minutes to absorb the supernatant. By adding 70% alcohol, the DNA is separated and precipitated from the mixture, and washed twice. After drying for 10 minutes, double distilled water was added, fully vortexed, and bathed in water at 65 °C for 3 minutes. After standing on a magnetic frame for 2 minutes, the supernatant was rich in DNA.

DNA size of 350 bp were generated using the Covaris E210 Ultrasonicator (Woburn, MA). A sequencing library was constructed with DNBSEQ-T7RS. The library was sequenced with BGI’ PCR DNBSEQ™ (Shenzhen, China). Raw data was filtered by fastp (Chen et al., 2018). Clean data were mapped to the reference genome using BWA-MEM version 0.7.17 (Li, 2013). The BWA-MEM algorithm was robust to sequencing errors and was suitable for a wide range of sequence lengths from 70 bp to several megabases. Sort by SAMtools version 1.10 (Li et al., 2009). Using Picard version 2.0.1 (http://broadinstitute.github.io/picard/)), you removed duplicate reads. Sequence variation detection and SNP detection were performed using GATK 4.17 version functions HaplotypeCaller and SelectVariants (McKenna et al., 2010). SNP was invoked using parameters”QD < 2.0, MQ < 40.0, FS > 60, SOR > 3.0, MQRankSum < − 12.5, ReadPosRankSum < − 8.0.” VCFtools version 1.16 (Li, 2013) was used to filter SNPs using parameters”max-missing 0.1, maf 0.05, maxDP 50 and minDP 10.”The identification of SNPs was based on the criteria of secondary allele frequency ≥ 0.05 and missing data rate ≥ 10% in the population. A total of 6,094,317 SNPs were identified on chromosome 1-10. The genetic relationship matrix (K) was generated using EMMAX (Kang et al., 2010) and used for GWAS analysis of Q matrices calculated using STRUCTURE 2.3.4 (Pritchard et al., 2000) as covariates.

The induction rate, embryogenic callus rate, browning rate and differentiation rate of mature seeds from all the 236 sorghum varieties under the same culture conditions were calculated. The GWAS mixed linear model (MLM) was used with the population structure as covariate, and the genetic relationship matrix was added as a random effect (Upadhyaya et al, 2022). For MLM (Q + K), the Bonferroni threshold of the significantly associated marker is calculated as -log10 (*p*) >= 8.1 (Upadhyaya et al, 2022).Manhattan maps were drawn by R package CMplot (Yin et al, 2021). Genes closest to or containing the linked SNPs are considered candidate genes based on the reference genome *Sorghum bicolor* v3.1.1 curated in Phytozome 13 (https://phytozome-next.jgi.doe.gov/). When reported candidate genes, we used Phytozome 13 to identify candidate genes closest from linked SNP markers. This ranges from 0-75 kb.Orthologs were identified as the best match by protein sequence BLAST in Phytozome 13.The default parameter settings were used for CMplot and BLAST.

## 3 Results

### 3.1 Phenotypic analysis

In the process of tissue culture of sorghum mature seeds, we found that some varieties had no callus induction ability, and/or the generated callus appeared to be water-soaked and could not become embryogenic calli, and/or low callus differentiation rate and browned. Regeneration-competent calli were granular compact yellowish as previously described (Belide et al., 2017).

We evaluated the callus induction rate, embryonic callus rate, browning rate and differentiation rate of mature seeds from the 236 sorghum varieties. Among these, 116 varieties were able to grow calli with an induction rate ranging from 1.1% to 68.97%. Out of these 116 varieties, 74 exhibited browning, producing brown phenolic substances with a browning rate ranging from 9% to 100%. There were 51 varieties whose callus tissues were embryonic, with an embryonic callus rate ranging from 9% to 100% (**Table 1**).Through statistical analysis of phenotypic data, we found that the induction rate, embryonic callus rate and differentiation rate basically conform to the normal distribution, while the browning rate does not conform to the normal distribution (**Supplementary Figure S1**).

**Table 1:** The average, range and standard deviation of different traits in 236 sorghum germplasm.

Character	Average (%)	Range (%)	SD
Induction rate	11.68	11-68.97	0.1513
Embryogenic callus rate	9.80	9-100	0.2141
Browning rate	17.14	5-100	0.3101
Differentiation rate	4.75	0-64.70	0.1131

**Figure 1:**
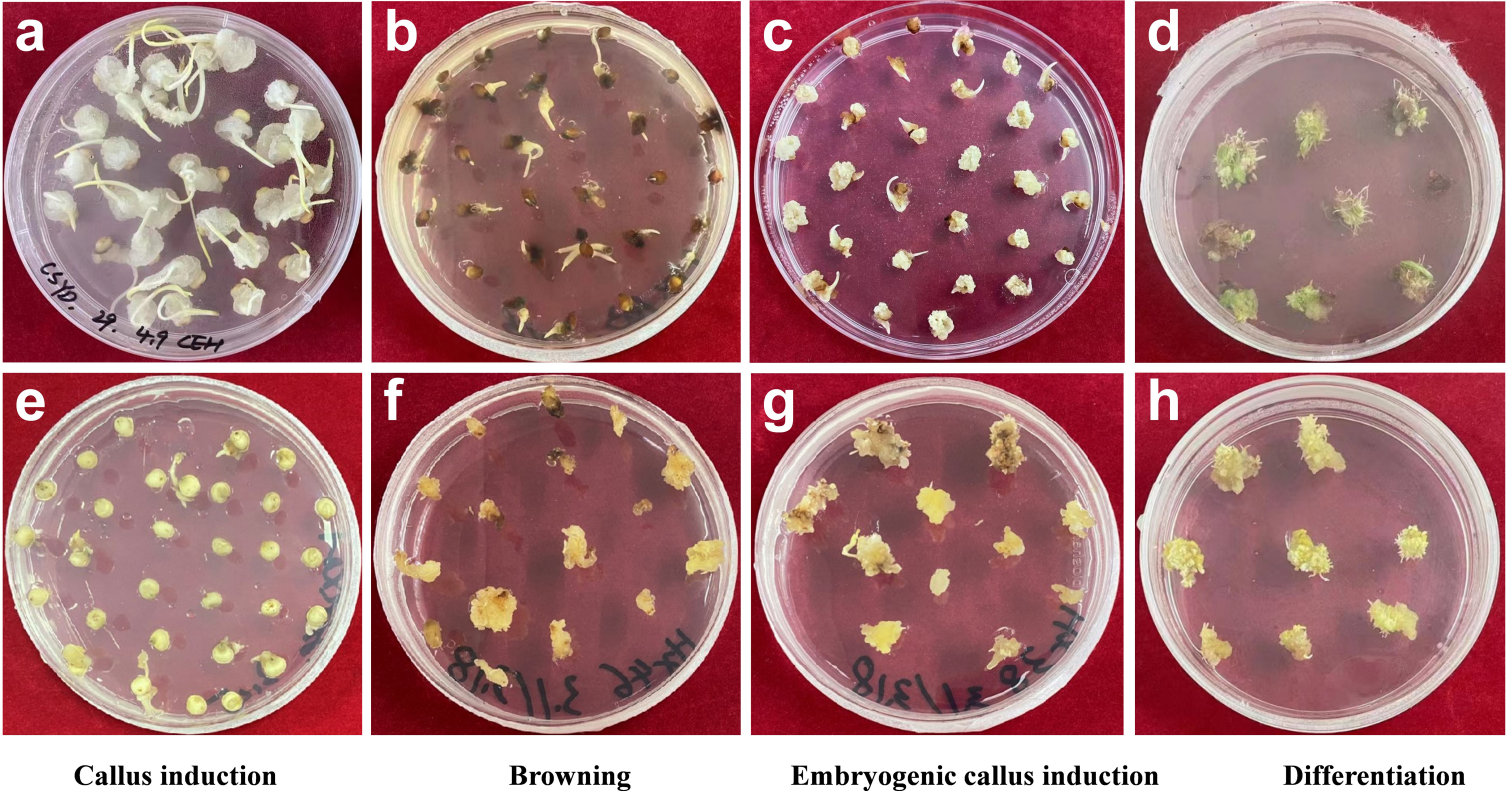
Examples of high/low callus induction rate **(a, e)**, browning rate **(b, f)**, embryonic callus rate **(c, g)**, and differentiation rate **(d, h)**.The corresponding varieties of a, b, c, d, e, f, g, h are IS4581, IS29233, IS7250, IS4698, IS608, IS7310, IS5667, IS2205, respectively.

After evaluating these varieties and based on high induction and differentiation rate but low browning rate, we identified five varieties which were efficient in plantlet regeneration using tissue culture (**Table 2**). This regeneration efficiency was not related to either seed coat color, agronomic types (races), or country of origin (**Table 2**). This agrees with a previous study using eight sorghum varieties of various seed coat color, countries of origin and agronomic races (Flinn et al., 2020). The examples of high/low callus induction rate, embryonic callus rate, browning rate and differentiation rate are presented in **Figure 1**.

**Table 2:** Top five sorghum varieties identified in this study for embryogenesis.

Variety ID	Induction rate	Embryogenic callus rate	Browning rate	Differentiation rate	Seed coat color	Agronomic types	Country of origin
IS5667	0.2833	0.1765	0.5294	0.647	Yellow	Durra	India
IS24503	0.067	0.5	0.5	0.5	Black	Bicolor	South Africa
IS8348	0.4	0.272	0.364	0.455	Red	Durra	Pakistan
IS4698	0.9324	0.5647	0.1486	0.3559	Pale yellow	Durra	India
IS5295	0.5	0.667	0.333	0.333	Pale yellow	Guinea	India

### 3.2 Association analysis

**Figure 2:**
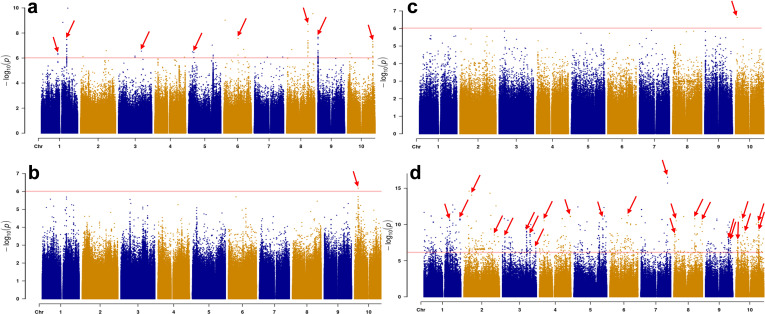
Manhattan plot for induction rate **(a)**, browning rate **(b)**, embryogenic callus rate **(c)** and differentiation rate **(d)**. Arrows indicate loci mapped to the four traits on each sorghum chromosome. Horizontal lines indicate the threshold -log(*p*) value.

Manhattan plots for callus induction rate, embryonic callus rate, browning rate and differentiation rate are presented in **Figure 2**. To identify quantitative trait loci (QTLs) underlying the traits, we sought for loci with -log(*p*) value above the Bonferroni but found no loci mapped in browning and embryonic callus rate. We decided to use a -log(*p*) value of 6.0 (Nguyen et al., 2020). With this value and that the linkage had to be with multiple markers as in previous studies (Upadhyaya et al., 2022; Wang et al., 2021b; Wang et al., 2024), we identified eight loci for callus induction rate, one locus each for browning and embryogenic callus rate, and 24 for differentiation rate (**Figure 2**). Detailed locus distribution and position on each chromosome are listed in **Table 3**.

**Table 3:** Significant SNPs linked to induction rate (a), browning rate (b), embryogenic callus rate (c), and differentiation rate (d) and candidate genes.

a. Significant SNP and linkage candidate genes associated with induction rate.										
SNP Code		Linked SNP		log(p) value		Candidate gene	Candidate gene position	Candidate gene annotation		Arabidopsis ortholog
CIR1-2		1:56522326		7.06195514		Sobic.001G288700	Chr01:56520646.56533452 forward	armadillo domain protein		AT5G37290
		1:56526473		7.4837751		Sobic.001G288800	Chr01:56540179.56543697 forward	60S RIBOSOMAL EXPORT PROTEIN NMD3		AT2G03820
CIR8-1		8:47287922		7.22652752		Sobic.008G099300	Chr08:47212476.47217111 reverse	cysteine-rich RLK(RECEPTOR-like protein kinase)		AT4G21410
CIR9-1		9:228936		7.11181749		Sobic.009G002700	Chr09:219056.221238 forward	serine protease inhibitor		AT1G47710
CIR10-1		10:56430182		7.05282567		Sobic.010G221800	Chr10:56433550.56437233 reverse	Phosphoglycerate kinase		AT1G79550
		10:56440210		6.46394932		Sobic.010G221900	Chr10:56438877.56439819 reverse	BTB-POZ and MATH domain 2/BPM2		AT3G06190
b. Significant SNP and linkage candidate genes associated with browning rate.										
SNP Code		Linked SNP		log(p) value		Candidate gene	Candidate gene position	Candidate gene annotation		Arabidopsis ortholog
BR10-1			10:7765278	6.18627689		Sobic.010G089700	Chr10:7785991.7790247 reverse	ethylene-responsive transcription factor RAP2-13/WIND1	AT1G78080	
c. Significant SNP and linkage candidate genes associated with embryogenic callus rate										
SNP Code		Linked SNP		log(p) value		Candidate gene	Candidate gene position	Candidate gene annotation		Arabidopsis ortholog
ECR10-1	10:3787773			6.62698218		Sobic.010G048900	Chr10:3783735.3784554 reverse	unknown		Not found/sorghum-specific
d. Significant SNP and linkage candidate genes associated with differentiation rate.										
SNP Code		Linked SNP		log(p) value		Candidate gene	Candidate gene position	Candidate gene annotation		Arabidopsis ortholog
DR1-1			1:56192501		9.68469579	Sobic.001G286700	Chr01:56194084.56200997 forward	homeobox-leucine zipper protein ROC3/HDG5		AT5G46880
DR1-2			1:78754383		10.7413553	Sobic.001G522600	Chr01:78750597.78763175 reverse	RING/FYVE/PHD zinc finger protein/histone acetyltransferase		AT2G19260
DR4-2			4:67438762		11.1123637	Sobic.004G344200	Chr04:67442361.67451659 forward	CLIP-associating protein-like		AT2G20190
DR7-1			7:58323967		9.93526404	Sobic.007G151300	Chr07:58321850.58322842 reverse	Cupin domain		AT1G72610
			7:58323968		9.93526404	Sobic.007G151400	Chr07:58341776.58345411 reverse	cytokinin dehydrogenase 11		AT5G21482
DR8-3			8:45553511		8.70245634	Sobic.008G096200	Chr08:45490278.45495182 reverse	serine/threonine-protein kinase D6PKL1		AT3G52890
DR8-4			8:62071567		9.02613902	Sobic.008G186100	Chr08:62072507.62078284 forward	transcription factor NAI1-like/bHLH		AT4G37850
DR9-2			9:54056760		7.79207878	Sobic.009G188100	Chr09:54039171.54047069 forward	receptor-like serine/threonine-protein kinase SD1-8		AT4G21380
DR10-4			10:49468869		10.3190549	Sobic.010G167500	Chr10:49525036.49529166 forward	Receptor protein-tyrosine kinase		AT5G25930
DR10-5			10:50398892		7.04949066	Sobic.010G171600	Chr10:50370480.50383361 forward	MITOGEN-ACTIVATED KINASE KINASE KINASE		AT4G08500

(Only important SNPs, the remaining SNPs in **Supplementary Table S4**).

In total, eight callus induction loci were mapped onto seven chromosomes with one each on chromosomes 3, 5, 6, 8, 9, 10 and two on chromosome 1. One locus was mapped each for callus browning and embryogenic callus induction on chromosome 10. Twenty-four differentiation loci were mapped for differentiation rate, with one each to chromosomes 5, 6, and 7, two each to chromosomes 1, 2, 4, and 9, four each to chromosomes 3 and 8, and five to chromosome 10 (**Figure 2**; **Table 3**). The *DR1-2* locus contained the tightest linked markers with an average -log(*p*) of 10.69 from six markers (**Table 3**).

### 3.3 Candidate gene identification

**Figure 3:**
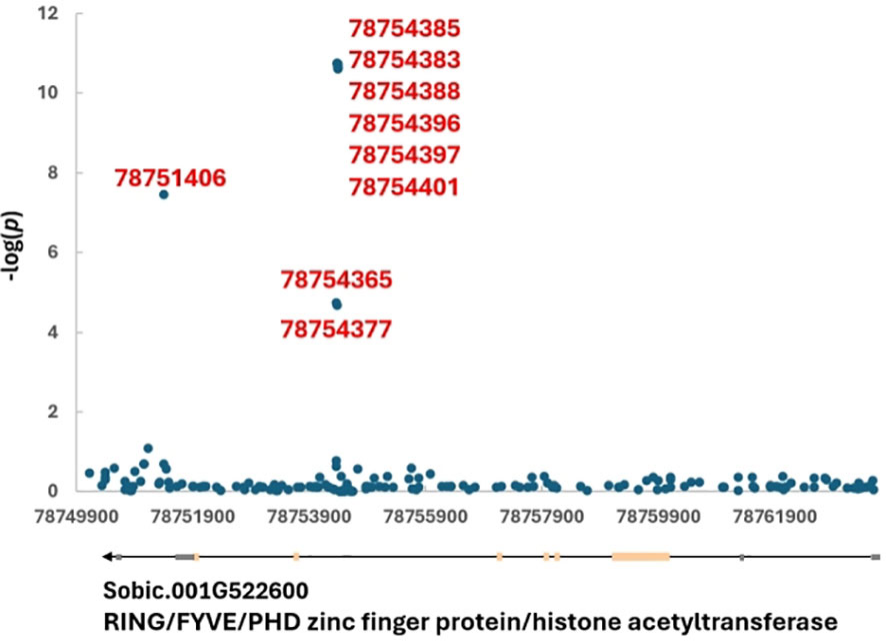
*DR1-2* locus and its candidate gene, Sobic.001G522600 coding for RING/FYVE/PHD zinc finger protein/histone acetyltransferase on chromosome 1. The linked SNP positions are listed.

To identify candidate gene for each locus, either gene(s) encompassing linked SNPs, closest or flanking genes were listed in **Table 3**. In total, 47 candidate genes were found from the 34 loci. To break it down, one candidate gene was found for 22 of the 34 mapped loci while two flanking genes were found for nine and 0, 3, and 4 genes were each found in one locus (**Table 3**). Six of the 22 loci (*CIR6-1*, *ECR10-1*, *DR2-1*, *DR4-1*, *DR6-1*, and *DR8-2*) contained a gene with unknown function and specific to sorghum - no Arabidopsis ortholog was found. *CIR1-1* and *CIR5-1* housed ZINC FINGER CCHC DOMAIN and AUXIN-REGULATED GENE INVOLVED IN ORGAN SIZE, respectively, and neither ortholog were found in Arabidopsis (**Table 3**). **Figure 3** shows the *DR1-2* locus and its candidate gene, Sobic.001G522600 coding for RING/FYVE/PHD zinc finger protein/histone acetyltransferase. Among all seven significant SNPs six were located in the 6^th^ intron and one in the last intron (the 8^th^) (**Figure 3**). Based on studies of their orthologs in Arabidopsis, quite a few our candidate genes play roles in embryogenesis which are further detailed in the Discussion.

## 4 Discussion

In this study, we set out to find highly competent sorghum varieties for genetic transformation and to understand the genetic architecture of embryogenesis. We have found five highly competent sorghum varieties for genetic transformation. They are IS5667, IS24503, IS8348, IS4698, and IS5295 (**Table 2**). These varieties will be valuable to future sorghum transgenic research. We mapped 34 QTLs to the four traits and identified 47 candidate genes, suggesting a complex mechanism underlying embryogenesis. Fourteen of the candidate genes are known to play roles in tissue culture and plant embryonic regeneration in other species. These are discussed in the following sections and will aid our understanding of the genetic mechanism behind embryogenesis.

Previous studies research on callus regeneration ability has primarily focused on plants such as Arabidopsis, rice, wheat, and maize and have identified callus formation and embryogenesis related candidate genes (Tuskan et al., 2018; Nagle et al., 2024; Zhang et al., 2019; Ma et al., 2018) However, almost all candidate genes identified in each study are unique suggests that a large number of genes are involved in the processes. Since overexpressing *WUSCHEL-related homeobox 2* (*WOX2*) transcription factor in maize increased transformation frequencies in previously nontransformable maize inbred lines (Lowe et al., 2016), we examined transcription factors reported by each study using Arabidopsis orthologs to compare across the studies. While AT1G03800 and AT3G14230 [both coding for ethylene response factor (ERF)/Apetala (AP) subfamily] are identified in rice (Zhang et al., 2019), in poplar (Nagle et al., 2024) they are AT3G12250 (TGA6), AT5G35550 (R2R3 MYB), At1g75250 (RADIALIS-LIKE SANT/MYB 3), AT4G16110 (a pollen-specific transcription factor), and AT4G27950 (another ERF/AP) and in maize (Ma et al., 2018) AT5G59340 (WOX2), AT1G62310 (JIMONJI 21), AT5G50670 (SBP 18), AT5G61620 (MYB 32), AT4G04890 (HDG2) and AT1G09530 (bHLH 43) are reported. These are 13 transcription factors reported in one eudicot and two monocots with two in rice, five in poplar and six in maize. Yet there is no transcription factor found in more than one study. In this study, we identified three transcription factors: AT1G78080 [WIND1 (ERF/AP), Sobic.010G089700], AT5G46880 (HDG5, Sobic.001G286700) and AT4G37850 (bHLH, Sobic.008G186100) (**Table 3**). Again, there is no overlap with genes discovered in poplar (Nagle et al., 2024), rice (Zhang et al., 2019) and maize (Ma et al., 2018). We downloaded the top 50 coexpressed genes of each sorghum transcription factor from Sorghum bicolor v3.1.1 at Phytozome 13 (McCormick et al., 2018) and found that while WIND1 (WOUND INDUCED DEDIFFERENTIATION 1) is coexpressed with seven other transcription factors (no homeobox genes) and two transporters (**Supplementary Table S5**), HDG5 is coexpressed with 14 other transcription factors (five of them homeobox genes) and also two transporters (**Supplementary Table S6**), but bHLH is coexpressed with five other transcription factors (no homeobox genes) and 11 transporters (**Supplementary Table S7**).This non-overlap in transcription factors during embryogenesis of different species could be due to two reasons. One is that the combination of species and tissue culture environments such as temperature, light, and medium composition could require different genes for embryogenesis. The other reason could be that embryogenesis starts a new plant and there are many transcription factors involved (Umehara et al., 2007; Yuan et al., 2024), as indicated by many coexpressed ones in our study, and only a few are mapped.

As in the case of overexpressing *WOX2* increasing maize transformation frequencies (Lowe et al., 2016), transcription factors play an essential role in embryogenesis. Arabidopsis WIND1 (WOUND INDUCED DEDIFFERENTIATION 1) is an ERF/AP transcription factor and is rapidly induced at the wound site, promoting cell dedifferentiation and subsequent cell proliferation to form a mass of pluripotent cells termed callus (Iwase et al., 2011). Ectopic expression of *WIND1* can bypass both wounding and auxin pre-treatment and increase *<i>de novo*</i> shoot regeneration from root explants cultured on shoot-regeneration promoting media (Iwase et al., 2015), suggesting that WIND1 can reprogram somatic cells to be pluripotent. WIND1 promotes callus formation and shoot regeneration by directly binding the promoter the *ENHANCER OF SHOOT REGENERATION1* (*ESR1-*AT1G12980) gene, which encodes another AP2/ERF transcription factor in *Arabidopsis thaliana* (Iwase et al., 2017). Therefore, WIND1 from *BR10-1* could promote regeneration and reduce callus browning rate in sorghum.

The four ERF/AP transcription factors (two from rice, WIND1 and ESR1) are potentially all involved in regeneration. It turns out that the BPM1 and BPM2 identified in *CIR10-1* (AT5G19000 and AT3G06190; **Table 3**) assemble with CUL3-based E3 ligases using their BTB/POZ domains (Weber et al., 2005; Gingerich et al., 2005). BPM proteins can also assemble/interact with members of the ERF/AP transcription factor family (Weber and Hellmann, 2009) and the assembled CUL3-based E3 ligases interact with a broad range of ERF/AP2 transcription factors, mediated by MATH-BTB/POZ proteins (Chen et al., 2013). The assembly with an E3 ligase causes degradation of the ERF/AP2 transcription factors via the 26S proteasome and loss of MATH-BTB/POZ proteins significantly affects plant development (Chen et al., 2013). All these have consequences for embryogenesis.

Other candidate genes related to embryogenesis are also found. A locus with the most tightly linked markers landed in the introns of Sobic.001G522600 which encodes RING/FYVE/PHD zinc finger protein/histone acetyltransferase. Both Sobic.001G522600 and AT2G19260 (its Arabidopsis ortholog) proteins contain an ELM2 domain towards their C-terminal (**Supplementary Figure S2**). ELM2 domain has been shown to recruit histone deacetylase (HDAC) 1 in humans (Ding et al., 2003). Histone deacetylation can prevent the initiation of somatic embryogenesis. Inhibiting HDAC by using an HDAC inhibitor, trichostatin A (TSA), triples embryogenic callus size in sorghum although it does not change embryogenic callus rate (Wu et al., 2024). TSA treatment also has been shown to repress seedling growth but upregulate expression of embryo-specific transcription factors such as LEC1 (Tanaka et al., 2008) which initiates formation of embryo-like structures in vegetative cells in *Arabidopsis* (Lotan et al., 1998).

Some candidate genes may perform cellular maintenance related functions. Sobic.001G288800 in *CIR1-2* encodes 60S RIBOSOMAL EXPORT PROTEIN NMD3 which in Arabidopsis participates in ribosome assembly and nuclear export of ribosomal subunits (Chen et al., 2012) and ribosome biogenesis is a fundamental process that provides cells with molecular factories for cellular protein production (Kressler et al., 2010). The ortholog of Sobic.009G002700 from *CIR9-1*, AT1G47710, encodes a serine protease inhibitor (AtSerpin1) which inhibits AtMC9 activity *in vitro* (Vercammen et al., 2006). AtMC9 can target and destroy multiple transcription and translation elongation factors, actins, ribosomal proteins (Tsiatsiani et al., 2013), all important players in basic cellular maintenance.

Some candidate genes are related to hormone-activated cell division. The ortholog of Sobic.004G344200 from *DR4-2*, AT2G20190, encodes a CLIP-associated protein (CLASP). In Arabidopsis CLASP promotes microtubule stability during mitosis, hence involved in cell division (Ambrose et al., 2007). Mutation in *CLASP* produces dwarf plants and significantly reduces cell number in cell division zone (Ambrose et al., 2007). The ortholog of Sobic.008G096200 from *DR8-3* (Arabidopsis ortholog: AT3G52890) is paralogous to Sobic.006G067700 (*Dw2*/*SbKIPK*), belonging to the AGCVIII subgroup of the AGC protein kinase family (Hilley et al., 2017). Known members of the AGCVIII subgroup have been reported to regulate auxin efflux transporters (Barbosa and Schwechheimer, 2014). AT3G52890 protein KIPK (KCBP-interacting protein kinase) interacts with the myosin tail region of KCBP (kinesin-like calmodulin-binding protein) (Day et al., 2000) which is known to be involved in cell division through its interaction with microtubules (Narasimhulu et al., 1997). Another hormone related gene is Sobic.007G151400. The ortholog of Sobic.007G151400 from *DR7-1*, AT5G21482, encodes cytokinin oxidase (CKX 7) which is expressed in the mature embryo sac and catalyzes the degradation of cytokinins (Köllmer et al., 2014).It is not clear if Sobic.008G096200 and Sobic.007G151400 impact the balance of auxin and cytokinin whose ratios determine how regeneration occurs (Ikeuchi et al., 2016).

We found three receptor protein kinases, Sobic.010G167500, Sobic.008G099300, and Sobic.009G188100 and a mitogen-activated kinase kinase kinase in Sobic.010G171600 (MAP KKK) (**Table 3**). A receptor-like protein kinase was identified as a major quantitative trait locus (QTL) that affects shoot regeneration in Arabidopsis (Motte et al., 2014). A nonsense mutation (*rpk1-5*) in the gene produces a protein truncated after the first 1/3 of LRR and completely cuts off the last 2/3 of LRR, transmembrane and kinase domains (Nodine et al., 2007). The mutant is almost completely recalcitrant to shoot regeneration (Motte et al., 2014). A MAP KKK was previously identified as one of the four callus formation genes in poplar (Tuskan et al., 2018).

In conclusion, in this study we evaluated 236 landrace varieties from the sorghum mini core collection and identified the top five varieties most amenable for *in vitro* embryogenesis and consequently genetic transformation. Using the results for GWAS allows us to map eight callus induction loci, one each for callus browning and embryogenic callus induction, and 24 differentiation loci, identified 47 candidate genes from the loci and provided evidence from the literature that orthologs for 14 of the genes play roles in cellular function and embryogenesis. Future projects will focus on functional characterization of selected genes and utilization of the five sorghum varieties for genetic transformation studies with TSA.

## Data Availability

The datasets presented in this study can be found in online repositories. The names of the repository/repositories and accession number(s) can be found in the article/**Supplementary Material**.
