# Supplementary figures and images for: Association mapping and identification of candidate genes for callus induction and regeneration using sorghum mature seeds
Source: Front Plant Sci. 2025 Apr 24;16:1430141. doi: 10.3389/fpls.2025.1430141 (PMC12058750; doi:10.3389/fpls.2025.1430141)

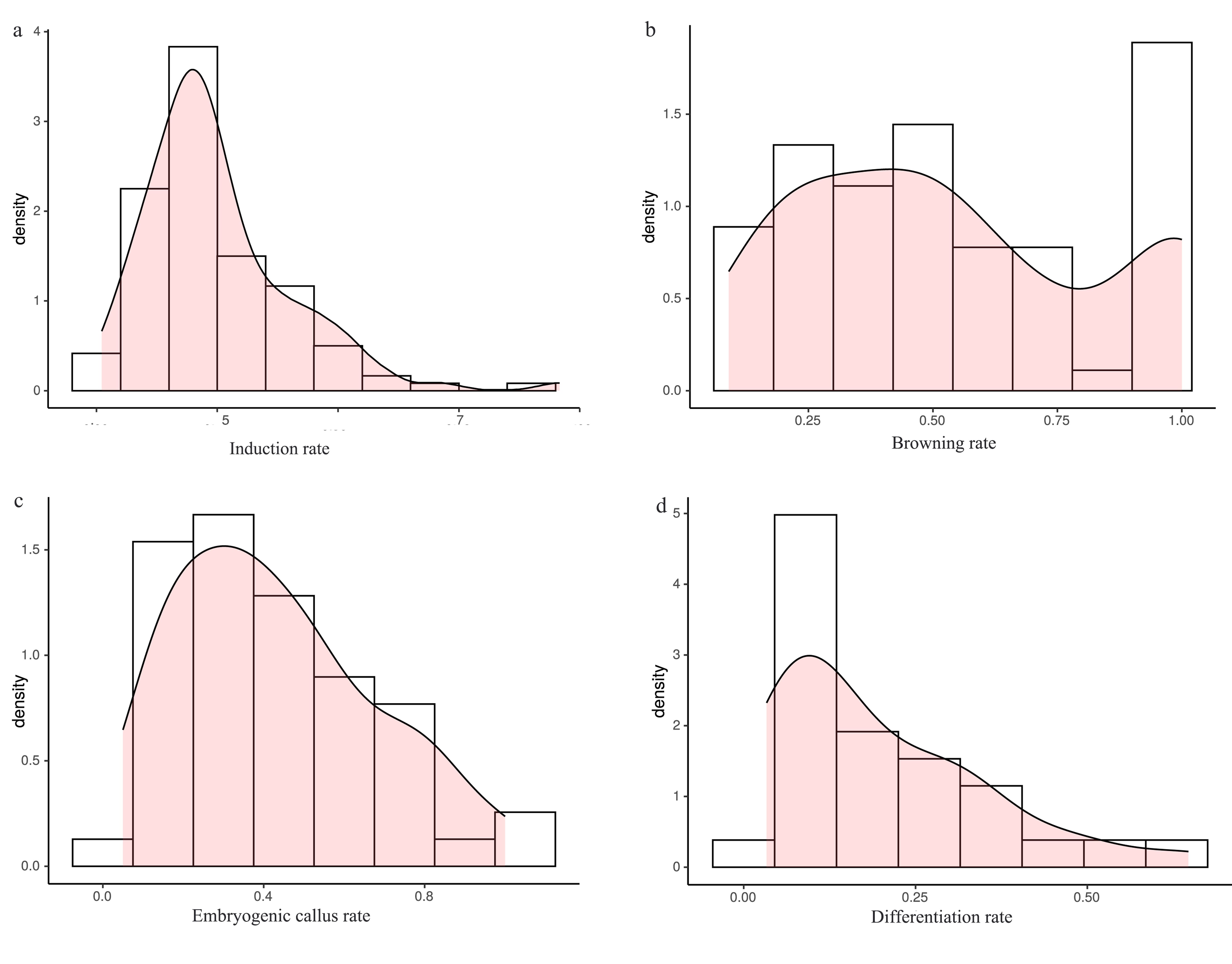

Supplement: Supplementary file 1 [file Image1.jpeg]

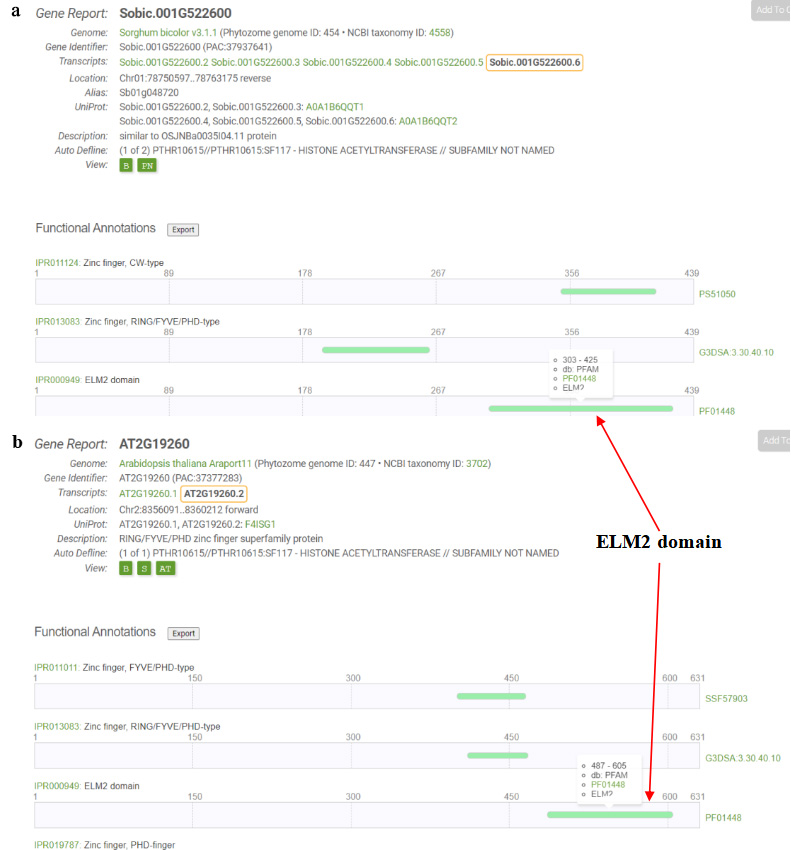

Supplement: Supplementary file 2 [file Image2.jpeg]
