# Supplementary material for: Association mapping and identification of candidate genes for callus induction and regeneration using sorghum mature seeds
Source: Front Plant Sci. 2025 Apr 24;16:1430141. doi: 10.3389/fpls.2025.1430141 (PMC12058750; doi:10.3389/fpls.2025.1430141)
Supplement: Supplementary file 3 [file Table1.docx]

Table S1 236 sorghum accessions and their origins

| S.NO | Accessions | Source  country | S.NO | Accessions | | Source  country | S.NO | Accessions | Source  country |
| --- | --- | --- | --- | --- | --- | --- | --- | --- | --- |
| 1 | IS473 | USA | 80 | IS13919 | South Africa | | 159 | IS26617 | Madagascar |
| 2 | IS602 | USA | 81 | IS13971 | South Africa | | 160 | IS26694 | South Africa |
| 3 | IS603 | USA | 82 | IS14010 | South Africa | | 161 | IS26701 | South Africa |
| 4 | IS608 | USA | 83 | IS14090 | Argentins | | 162 | IS26737 | South Africa |
| 5 | IS995 | USA | 84 | IS14290 | Botswana | | 163 | IS26749 | South Africa |
| 6 | IS1004 | India | 85 | IS14779 | Cameroon | | 164 | IS27034 | Sudan |
| 7 | IS1041 | India | 86 | IS14861 | Cameroon | | 165 | IS27557 | Burkina Faso |
| 8 | IS1212 | China | 87 | IS15170 | Cameroon | | 166 | IS27697 | Sierra Leone |
| 9 | IS1219 | China | 88 | IS15478 | Cameroon | | 167 | IS27786 | Morocco |
| 10 | IS1233 | China | 89 | IS15744 | Cameroon | | 168 | IS27887 | South Africa |
| 11 | IS2205 | India | 90 | IS15931 | Cameroon | | 169 | IS27912 | South Africa |
| 12 | IS2379 | South Africa | 91 | IS15945 | Cameroon | | 170 | IS28141 | Yemen,Republic of |
| 13 | IS2382 | South Africa | 92 | IS16151 | Cameroon | | 171 | IS28313 | Yemen,Republic of |
| 14 | IS2389 | South Africa | 93 | IS16528 | Cameroon | | 172 | IS28389 | Yemen,Republic of |
| 15 | IS2397 | South Africa | 94 | IS17941 | India | | 173 | IS28449 | Yemen,Republic of |
| 16 | IS2413 | Iran | 95 | IS17980 | India | | 174 | IS28451 | Yemen,Republic of |
| 17 | IS2426 | Afghanistan | 96 | IS18039 | India | | 175 | IS28614 | Yemen,Republic of |
| 18 | IS2864 | South Africa | 97 | IS19153 | Ethiopia | | 176 | IS28747 | Yemen,Republic of |
| 19 | IS2872 | Egypt | 98 | IS18758 | Sudan | | 177 | IS28849 | Yemen,Republic of |
| 20 | IS2902 | Nigeria | 99 | IS19262 | Sudan | | 178 | IS29091 | Yemen,Republic of |
| 21 | IS3121 | Kenya | 100 | IS19389 | Bangladesh | | 179 | IS29100 | Yemen,Republic of |
| 22 | IS3158 | South Africa | 101 | IS19445 | Botswana | | 180 | IS29187 | Swaziland |
| 23 | IS3971 | India | 102 | IS19450 | Botswana | | 181 | IS29233 | Swaziland |
| 24 | IS4060 | India | 103 | IS19676 | Zimbabwe | | 182 | IS29239 | Swaziland |
| 25 | IS4092 | India | 104 | IS19859 | India | | 183 | IS29241 | Swaziland |
| 26 | IS4360 | India | 105 | IS19975 | Senegal | | 184 | IS29269 | Swaziland |
| 27 | IS4372 | India | 106 | IS20195 | Niger | | 185 | IS29304 | Swaziland |
| 28 | IS4515 | India | 107 | IS20625 | USA | | 186 | IS29314 | Swaziland |
| 29 | IS4581 | India | 108 | IS20632 | USA | | 187 | IS29326 | Swaziland |
| 30 | IS4613 | India | 109 | IS20679 | USA | | 188 | IS29335 | Swaziland |
| 31 | IS4631 | India | 110 | IS20697 | USA | | 189 | IS29358 | Lesotho |
| 32 | IS4698 | India | 111 | IS20727 | USA | | 190 | IS29392 | Lesotho |
| 33 | IS4951 | India | 112 | IS20740 | USA | | 191 | IS29441 | Lesotho |
| 34 | IS5094 | India | 113 | IS20743 | USA | | 192 | IS29468 | Lesotho |
| 35 | IS5295 | India | 114 | IS20816 | USA | | 193 | IS29519 | Lesotho |
| 36 | IS5301 | India | 115 | IS20956 | Indonesia | | 194 | IS29565 | Lesotho |
| 37 | IS5386 | India | 116 | IS21083 | Kenya | | 195 | IS29568 | Lesotho |
| 38 | IS5667 | India | 117 | IS21512 | Malawi | | 196 | IS29582 | Lesotho |
| 39 | IS5919 | India | 118 | IS21645 | Malawi | | 197 | IS29606 | South Africa |
| 40 | IS6351 | India | 119 | IS21863 | Syrian Arab Repubilic | | 198 | IS29627 | South Africa |
| 41 | IS6354 | India | 120 | IS22239 | Botswana | | 199 | IS29654 | China |
| 42 | IS6421 | India | 121 | IS22294 | Botswana | | 200 | IS29689 | Zimbabwe |
| 43 | IS7131 | Uganda | 122 | IS22609 | Sri Lanka | | 201 | IS29714 | Zimbabwe |
| 44 | IS7250 | Nigeria | 123 | IS22616 | MYA | | 202 | IS29733 | Zimbabwe |
| 45 | IS7305 | Nigeria | 124 | IS22720 | Somalia | | 203 | IS29772 | Zimbabwe |
| 46 | IS7310 | Nigeria | 125 | IS22799 | Somalia | | 204 | IS29914 | Zimbabwe |
| 47 | IS7679 | Nigeria | 126 | IS22986 | Sudan | | 205 | IS29950 | Zimbabwe |
| 48 | IS7957 | Nigeria | 127 | IS23216 | Zambia | | 206 | IS30079 | Zimbabwe |
| 49 | IS7987 | Nigeria | 128 | IS23514 | Ethiopia | | 207 | IS30092 | Zimbabwe |
| 50 | IS8012 | Japan | 129 | IS23521 | Ethiopia | | 208 | IS30231 | Zimbabwe |
| 51 | IS8348 | Pakistan | 130 | IS23579 | Ethiopia | | 209 | IS30383 | China |
| 52 | IS8774 | South Africa | 131 | IS23586 | Ethiopia | | 210 | IS30400 | China |
| 53 | IS8777 | Uganda | 132 | IS23590 | Ethiopia | | 211 | IS30417 | China |
| 54 | IS8916 | Uganda | 133 | IS23644 | Gambia | | 212 | IS30443 | China |
| 55 | IS9108 | Kenya | 134 | IS23684 | Mozambique | | 213 | IS30450 | China |
| 56 | IS9113 | Kenya | 135 | IS23891 | Yemen,Republic of | | 214 | IS30451 | China |
| 57 | IS9177 | Kenya | 136 | IS23992 | Yemen,Republic of | | 215 | IS30460 | China |
| 58 | IS9745 | Sudan | 137 | IS24139 | Tanzania | | 216 | IS30466 | China |
| 59 | IS10302 | Thailand | 138 | IS24175 | Tanzania | | 217 | IS30507 | Korea,Repulic of |
| 60 | IS10757 | Chad | 139 | IS24218 | Tanzania | | 218 | IS30508 | Korea,Repulic of |
| 61 | IS10867 | Chad | 140 | IS24348 | India | | 219 | IS30533 | Korea,Repulic of |
| 62 | IS10969 | USA | 141 | IS24453 | South Africa | | 220 | IS30536 | Korea,Repulic of |
| 63 | IS11026 | Ethiopia | 142 | IS24462 | South Africa | | 221 | IS30562 | Korea,Repulic of |
| 64 | IS11473 | Ethiopia | 143 | IS24463 | South Africa | | 222 | IS30572 | Cameroon |
| 65 | IS11619 | Ethiopia | 144 | IS24492 | South Africa | | 223 | IS30838 | Cameroon |
| 66 | IS11919 | Ethiopia | 145 | IS24503 | South Africa | | 224 | IS31043 | Uganda |
| 67 | IS12302 | Zimbabwe | 146 | IS24939 | Zambia | | 225 | IS31186 | Uganda |
| 68 | IS12447 | Sudan | 147 | IS24953 | Zambia | | 226 | IS31446 | Uganda |
| 69 | IS12697 | Australia | 148 | IS25089 | Ghana | | 227 | IS31651 | Zaire |
| 70 | IS12706 | USA | 149 | IS25249 | Ethiopia | | 228 | IS31681 | Algeria |
| 71 | IS12804 | Turkey | 150 | IS25301 | Ethiopia | | 229 | IS31706 | Yemen,Republic of |
| 72 | IS12883 | India | 151 | IS25548 | Rwanda | | 230 | IS31714 | Yemen,Republic of |
| 73 | IS12937 | Ethiopia | 152 | IS25732 | Mali | | 231 | IS32245 | Yemen,Republic of |
| 74 | IS12945 | Nicaragua | 153 | IS25836 | Mali | | 232 | IS32439 | India |
| 75 | IS12965 | Cuba | 154 | IS25910 | Mali | | 233 | IS32787 | Somalia |
| 76 | IS13294 | Venezuela | 155 | IS26025 | Mali | | 234 | IS33023 | Tanzania |
| 77 | IS13549 | Mexico | 156 | IS26046 | Mali | | 235 | IS33090 | Honduras |
| 78 | IS13782 | South Africa | 157 | IS26222 | Togo | | 236 | IS33353 | Kenya |
| 79 | IS13893 | South Africa | 158 | IS26484 | Benin | |  |  |  |
|  |  |  |  |  |  | |  |  |  |
|  |  |  |  |  |  | |  |  |  |
|  |  |  |  |  |  | |  |  |  |
|  |  |  |  |  |  | |  |  |  |
|  |  |  |  |  |  | |  |  |  |
|  |  |  |  |  |  | |  |  |  |
|  |  |  |  |  |  | |  |  |  |
|  |  |  |  |  |  | |  |  |  |
|  |  |  |  |  |  | |  |  |  |
|  |  |  |  |  |  | |  |  |  |
|  |  |  |  |  |  | |  |  |  |
|  |  |  |  |  |  | |  |  |  |
|  |  |  |  |  |  | |  |  |  |
