# Supplementary material for: Association mapping and identification of candidate genes for callus induction and regeneration using sorghum mature seeds
Source: Front Plant Sci. 2025 Apr 24;16:1430141. doi: 10.3389/fpls.2025.1430141 (PMC12058750; doi:10.3389/fpls.2025.1430141)
Supplement: Supplementary file 4 [file Table2.docx]

Table S2 Culture medium formula

a. induction medium

| MS salt | 4.43g/L |
| --- | --- |
| Sucrose | 30g/L |
| Potassium dihydrogen phosphate | 1g/L |
| Inositol | 0.1g/L |
| Aspartic acid | 1g/L |
| L-proline | 1g/L |
| Nicotinic acid | 0.5mg/L |
| Glycine | 0.4mg/L |
| 2,4-D | 2mg/L |
| PH=5.7 | |
| Phytagel | 3.5g/L |
| High pressure steam sterilization, cooling to 55 degrees | |
| Pyridoxine HCL | 0.5mg/L |
| Thiamine HCL | 0.1mg/L |
| L-Ascorbic acid | 10mg/L |
| Cupric sulfate | 1.875mg/L |

b. subculture medium

| MS salt | 4.43g/L |
| --- | --- |
| Sucrose | 30g/L |
| Potassium dihydrogen phosphate | 1g/L |
| Inositol | 0.1g/L |
| Aspartic acid | 1g/L |
| L-proline | 1g/L |
| Nicotinic acid | 0.5mg/L |
| Glycine | 0.4mg/L |
| 2,4-D | 5mg/L |
| PH=5.7 | |
| Phytagel | 3.5g/L |
| High pressure steam sterilization, cooling to 55 degrees | |
| Pyridoxine HCL | 0.5mg/L |
| Thiamine HCL | 0.1mg/L |
| L-Ascorbic acid | 10mg/L |
| Cupric sulfate | 1.875mg/L |

c.differential medium

| MS salt | 4.43g/L |
| --- | --- |
| Sucrose | 30g/L |
| PH=5.7 | |
| Phytagel | 3.5g/L |
| High pressure steam sterilization, cooling to 55 degrees | |
| 6-Benzylaminopurine | 1mg/L |
| Indole-3-acetic acid | 1mg/ |
| copper sulphate | 1.25mg/L |
