# Supplementary material for: Association mapping and identification of candidate genes for callus induction and regeneration using sorghum mature seeds
Source: Front Plant Sci. 2025 Apr 24;16:1430141. doi: 10.3389/fpls.2025.1430141 (PMC12058750; doi:10.3389/fpls.2025.1430141)
Supplement: Supplementary file 5 [file Table3.docx]

Table S3 DNA extraction medium

a. DNA lysis solution

| KAC | 9.82g/L |
| --- | --- |
| EDTA | 18.61g/L |
| Guanidine hydrochloride | 477.65g/L |
| Sodium citrate | 1.4705g/L |
| Tween 20® | 20ml/L |
| PH=5.2 | |

b. DNA binding solution

| PEG8000 | 50g/L |
| --- | --- |
| GITC | 474.4g/L |
| Sodium chloride | 29.22g/L |
