# Supplementary material for: Association mapping and identification of candidate genes for callus induction and regeneration using sorghum mature seeds
Source: Front Plant Sci. 2025 Apr 24;16:1430141. doi: 10.3389/fpls.2025.1430141 (PMC12058750; doi:10.3389/fpls.2025.1430141)
Supplement: Supplementary file 6 [file Table4.docx]

Table S4 Significant SNPs linked to induction rate, browning rate, embryogenic callus rate, and differentiation and candidate genes.

|  | **SNP Code** | **Linked SNP** | **log(p) value** | **Candidate gene** | **Candidate gene position** | **Candidate gene annotation** | **Arabidopsis ortholog** |
| --- | --- | --- | --- | --- | --- | --- | --- |
| Callus induction rate | CIR1-1 | 1:36748223 | 6.28241797 | Sobic.001G261517 | Chr01:36788115..36789957 reverse | ZINC FINGER CCHC DOMAIN | Not found |
|  |  | 1:36748226 | 6.35859324 |  |  |  |  |
|  | CIR1-2 | 1:56522326 | 7.06195514 | Sobic.001G288700 | Chr01:56520646..56533452 forward | armadillo domain protein | AT5G37290 |
|  |  | 1:56526473 | 7.4837751 | Sobic.001G288800 | Chr01:56540179..56543697 forward | 60S RIBOSOMAL EXPORT PROTEIN NMD3 | AT2G03820 |
|  |  | 1:56529317 | 7.4837751 |  |  |  |  |
|  | CIR3-1 | 3:51062183 | 4.66559531 | Sobic.003G190100 | Chr03:51055764..51060854 forward | gamma vacuolar processing enzyme | AT4G32940 |
|  |  | 3:51062219 | 6.54867122 |  |  |  |  |
|  | CIR5-1 | 5:11555256 | 6.4372962 | Sobic.005G084600 | Chr05:11513753..11515603 forward | AUXIN-REGULATED GENE INVOLVED IN ORGAN SIZE | Not found |
|  |  | 5:11555283 | 6.03632355 |  |  |  |  |
|  | CIR6-1 | 6:31566696 | 6.23735194 | Sobic.006G044866 | Chr06:31568537..31570679 forward | unknown | Not found/sorghum-specific |
|  |  | 6:31566744 | 7.55552267 |  |  |  |  |
|  | CIR8-1 | 8:47287922 | 7.22652752 | Sobic.008G099300 | Chr08:47212476..47217111 reverse | cysteine-rich RLK(RECEPTOR-like protein kinase) | AT4G21410 |
|  |  | 8:47287923 | 8.69345062 |  |  |  |  |
|  |  | 8:47287925 | 8.13561951 |  |  |  |  |
|  |  | 8:47287985 | 7.39029796 |  |  |  |  |
|  | CIR9-1 | 9:228936 | 7.11181749 | Sobic.009G002700 | Chr09:219056..221238 forward | serine protease inhibitor | AT1G47710 |
|  |  | 9:231833 | 7.53665637 | Sobic.009G002800 | Chr09:224224..227090 reverse | STIP1 homology and U-box containing protein 1 | AT3G07370 |
|  |  | 9:239884 | 7.6291856 | Sobic.009G002900 | Chr09:228045..231444 reverse | RNASE H DOMAIN-CONTAINING PROTEIN | AT1G24090 |
|  |  | 9:242020 | 7.07867686 | Sobic.009G003000 | Chr09:232446..243556 reverse | Methionine S-methyltransferase | AT5G49810 |
|  | CIR10-1 | 10:56430182 | 7.05282567 | Sobic.010G221800 | Chr10:56433550..56437233 reverse | Phosphoglycerate kinase | AT1G79550 |
|  |  | 10:56440210 | 6.46394932 | Sobic.010G221900 | Chr10:56438877..56439819 reverse | BTB-POZ and MATH domain 2/BPM2 | AT3G06190 |
|  |  | 10:56441693 | 6.86860976 | Sobic.010G222000 | Chr10:56444547..56445812 reverse | BTB-POZ and MATH domain 1/BPM1 | AT5G19000 |
|  |  | 10:56442949 | 7.30436266 |  |  |  |  |
|  |  | 10:56443131 | 7.06413959 |  |  |  |  |
|  |  | 10:56445379 | 6.89485324 |  |  |  |  |
| Browning rate | BR10-1 | 10:7765278 | 6.18627689 | Sobic.010G089700 | Chr10:7785991..7790247 reverse | ethylene-responsive transcription factor RAP2-13/WIND1 | AT1G78080 |
|  |  | 10:7765355 | 5.45775627 |  |  |  |  |
| Embryogenic callus rate | ECR10-1 | 10:3787773 | 6.62698218 | Sobic.010G048900 | Chr10:3783735..3784554 reverse | unknown | Not found/sorghum-specific |
|  |  | 10:3787848 | 4.56157693 |  |  |  |  |
| Differentiation rate | DR1-1 | 1:56192501 | 9.68469579 | Sobic.001G286700 | Chr01:56194084..56200997 forward | homeobox-leucine zipper protein ROC3/HDG5 | AT5G46880 |
|  |  | 1:56192505 | 10.5855757 |  |  |  |  |
|  |  | 1:56192507 | 10.5855757 |  |  |  |  |
|  |  | 1:56192520 | 10.5855757 |  |  |  |  |
|  | DR1-2 | 1:78754383 | 10.7413553 | Sobic.001G522600 | Chr01:78750597..78763175 reverse | RING/FYVE/PHD zinc finger protein/histone acetyltransferase | AT2G19260 |
|  |  | 1:78754385 | 10.7480809 |  |  |  |  |
|  |  | 1:78754388 | 10.7364344 |  |  |  |  |
|  |  | 1:78754396 | 10.6734025 |  |  |  |  |
|  |  | 1:78754397 | 10.6370874 |  |  |  |  |
|  |  | 1:78754401 | 10.6082029 |  |  |  |  |
|  | DR2-1 | 2:66594371 | 7.70989136 | Sobic.002G286150 | Chr02:66593995..66595407 reverse | unknown | Not found/sorghum-specific |
|  |  | 2:66594805 | 8.5465444 |  |  |  |  |
|  |  | 2:66595515 | 7.64604682 |  |  |  |  |
|  | DR2-1 | 2:16763237 | 14.0395907 |  |  |  |  |
|  |  | 2:16763266 | 9.52271893 |  |  |  |  |
|  | DR3-1 | 3:5054251 | 8.19475478 | Sobic.003G056600 | Chr03:5054492..5054804 forward | histone H4 | AT2G28740 |
|  |  | 3:5054681 | 6.99497982 |  |  |  |  |
|  | DR3-2 | 3:53008803 | 8.92325534 | Sobic.003G201200 | Chr03:53020445..53021462 reverse | SMALL HEAT-SHOCK PROTEIN HSP20 | AT5G04890 |
|  |  | 3:53008852 | 7.80372008 | Sobic.003G201150 | Chr03:53004268..53006152 forward | ZINC FINGER CCHC DOMAIN CONTAINING PROTEIN | AT3G43590 |
|  | DR3-3 | 3:60344186 | 8.57419908 | Sobic.003G266500 | Chr03:60343803..60347353 forward | phosphoethanolamine N-methyltransferase | AT3G18000 |
|  |  | 3:60345749 | 8.57419908 | Sobic.003G266600 | Chr03:60350152..60356018 forward | phosphoethanolamine N-methyltransferase | AT3G18000 |
|  |  | 3:60346192 | 8.35809588 |  |  |  |  |
|  |  | 3:60346517 | 8.57419908 |  |  |  |  |
|  |  | 3:60346519 | 8.57419908 |  |  |  |  |
|  |  | 3:60346942 | 8.57419908 |  |  |  |  |
|  |  | 3:60346997 | 8.5566795 |  |  |  |  |
|  |  | 3:60347208 | 8.55523652 |  |  |  |  |
|  |  | 3:60347269 | 8.57419908 |  |  |  |  |
|  |  | 3:60347307 | 8.54466883 |  |  |  |  |
|  |  | 3:60347445 | 8.55523652 |  |  |  |  |
|  |  | 3:60348036 | 8.57419908 |  |  |  |  |
|  |  | 3:60348137 | 8.57419908 |  |  |  |  |
|  |  | 3:60348666 | 8.55523652 |  |  |  |  |
|  |  | 3:60348834 | 8.57419908 |  |  |  |  |
|  | DR3-4 | 3:72960343 | 6.70737249 | Sobic.003G425200 | Chr03:72967860..72968921 forward | EF-HAND CALCIUM-BINDING DOMAIN | AT1G05990 |
|  |  | 3:72961846 | 6.30814047 | Sobic.003G425100 | Chr03:72927879..72933126 reverse | protein OSB2, chloroplastic isoform X1 | AT4G20010 |
|  | DR4-1 | 4:10064678 | 7.62511314 | Sobic.004G106250 | Chr04:10063872..10067529 reverse | unknown | Not found/sorghum-specific |
|  |  | 4:10064682 | 7.62511314 |  |  |  |  |
|  |  | 4:10065207 | 8.51704796 |  |  |  |  |
|  |  | 4:10065272 | 10.5019447 |  |  |  |  |
|  | DR4-2 | 4:67438762 | 11.1123637 | Sobic.004G344200 | Chr04:67442361..67451659 forward | CLIP-associating protein-like | AT2G20190 |
|  |  | 4:67438773 | 7.1273334 |  |  |  |  |
|  |  | 4:67438781 | 11.1123637 |  |  |  |  |
|  |  | 4:67438785 | 11.1123637 |  |  |  |  |
|  |  | 4:67438791 | 7.1273334 |  |  |  |  |
|  |  | 4:67438799 | 11.1123637 |  |  |  |  |
|  | DR5-1 | 5:63338933 | 9.34902336 | Sobic.005G159400 | Chr05:63341933..63343870 forward | Patatin-like phospholipase | AT5G43590 |
|  |  | 5:63339339 | 9.26952772 |  |  |  |  |
|  |  | 5:63339443 | 10.7567624 |  |  |  |  |
|  |  | 5:63339489 | 9.37488999 |  |  |  |  |
|  |  | 5:63339651 | 9.34736017 |  |  |  |  |
|  | DR6-1 | 6:40272462 | 11.2124841 | Sobic.006G057600 | Chr06:40270790..40273561 forward | unknown | Not found/sorghum-specific |
|  |  | 6:40272484 | 8.04821856 |  |  |  |  |
|  |  | 6:40272490 | 8.04821856 |  |  |  |  |
|  |  | 6:40272509 | 8.04821856 |  |  |  |  |
|  | DR7-1 | 7:58323967 | 9.93526404 | Sobic.007G151300 | Chr07:58321850..58322842 reverse | Cupin domain | AT1G72610 |
|  |  | 7:58323968 | 9.93526404 | Sobic.007G151400 | Chr07:58341776..58345411 reverse | cytokinin dehydrogenase 11 | AT5G21482 |
|  |  | 7:58323971 | 15.7151294 |  |  |  |  |
|  |  | 7:58323986 | 16.531302 |  |  |  |  |
|  | DR8-1 | 8:2087138 | 8.61262692 | Sobic.008G023801 | Chr08:2092270..2098547 reverse | Trypsin-like serine proteases | AT5G27660 |
|  |  | 8:2088193 | 8.61262692 |  |  |  |  |
|  |  | 8:2088413 | 8.61262692 |  |  |  |  |
|  |  | 8:2089751 | 8.56015208 |  |  |  |  |
|  |  | 8:2091278 | 8.30572301 |  |  |  |  |
|  |  | 8:2091783 | 8.58926164 |  |  |  |  |
|  |  | 8:2094896 | 8.61262692 |  |  |  |  |
|  |  | 8:2100644 | 8.61262692 |  |  |  |  |
|  | DR8-2 | 8:4931003 | 10.4956615 | Sobic.008G050200 | Chr08:4926825..4928470 reverse | unknown | Not found/sorghum-specific |
|  |  | 8:4931016 | 9.20936237 |  |  |  |  |
|  | DR8-3 | 8:45553511 | 8.70245634 | Sobic.008G096200 | Chr08:45490278..45495182 reverse | serine/threonine-protein kinase D6PKL1 | AT3G52890 |
|  |  | 8:45553544 | 9.78096363 |  |  |  |  |
|  |  | 8:45576847 | 10.7719348 |  |  |  |  |
|  |  | 8:45576849 | 10.7719348 |  |  |  |  |
|  | DR8-4 | 8:62071567 | 9.02613902 | Sobic.008G186100 | Chr08:62072507..62078284 forward | transcription factor NAI1-like/bHLH | AT4G37850 |
|  |  | 8:62071602 | 10.4147575 |  |  |  |  |
|  | DR9-1 | 9:50534368 | 7.64547704 | Sobic.009G148200 | Chr09:50536574..50541986 forward | Deoxycytidine kinase | AT1G72040 |
|  |  | 9:50537363 | 7.74817288 |  |  |  |  |
|  |  | 9:50537398 | 7.72907559 |  |  |  |  |
|  |  | 9:50539454 | 7.31866991 |  |  |  |  |
|  |  | 9:50543633 | 7.3581704 |  |  |  |  |
|  |  | 9:50543765 | 7.54812435 |  |  |  |  |
|  |  | 9:50544314 | 7.3581704 |  |  |  |  |
|  | DR9-2 | 9:54051684 | 6.35078975 | Sobic.009G188300 | Chr09:54058558..54062690 forward | Universal stress protein | AT1G11360 |
|  |  | 9:54056760 | 7.79207878 | Sobic.009G188100 | Chr09:54039171..54047069 forward | receptor-like serine/threonine-protein kinase SD1-8 | AT4G21380 |
|  |  | 9:54058253 | 6.73326401 |  |  |  |  |
|  |  | 9:54058293 | 6.3788743 |  |  |  |  |
|  |  | 9:54058299 | 6.3788743 |  |  |  |  |
|  |  | 9:54058309 | 6.73326401 |  |  |  |  |
|  | DR10-1 | 10:4274020 | 6.84629885 | Sobic.010G054500 | Chr10:4273881..4275031 reverse | OXIDOREDUCTASE, 2OG-FE II OXYGENASE | AT2G30830 |
|  |  | 10:4274071 | 7.77000752 | Sobic.010G056200 | Chr10:4375339..4380759 forward | WD40 REPEAT PROTEIN | AT5G56190 |
|  |  | 10:4274093 | 6.65252331 |  |  |  |  |
|  |  | 10:4314439 | 6.89890691 |  |  |  |  |
|  |  | 10:4375346 | 7.73514158 |  |  |  |  |
|  | DR10-2 | 10:14598607 | 10.503715 | Sobic.010G123600 | Chr10:14628532..14636955 forward | WW domain-containing protein-like | AT3G13225 |
|  |  | 10:14598648 | 10.548605 | Sobic.010G123500 | Chr10:14532266..14537924 reverse | B-box zinc finger (zf-B_box) | AT5G48250 |
|  | DR10-3 | 10:19748855 | 7.34666741 | Sobic.010G134500 | Chr10:19639340..19667448 forward | 3'-5' EXONUCLEASE ERI1-RELATED | AT3G15140 |
|  |  | 10:19749035 | 8.85298526 |  |  |  |  |
|  |  | 10:19749064 | 7.28725298 |  |  |  |  |
|  |  | 10:19749078 | 7.31332148 |  |  |  |  |
|  | DR10-4 | 10:49468869 | 10.3190549 | Sobic.010G167500 | Chr10:49525036..49529166 forward | Receptor protein-tyrosine kinase | AT5G25930 |
|  |  | 10:49468871 | 10.3190549 |  |  |  |  |
|  | DR10-5 | 10:50395220 | 7.04652978 | Sobic.010G171800 | Chr10:50424293..50428647 forward | L-ascorbate oxidase | AT5G21105 |
|  |  | 10:50398892 | 7.04949066 | Sobic.010G171600 | Chr10:50370480..50383361 forward | MITOGEN-ACTIVATED KINASE KINASE KINASE | AT4G08500 |
|  |  | 10:50398901 | 7.06644292 |  |  |  |  |
|  |  | 10:50401004 | 8.53548514 |  |  |  |  |
|  |  | 10:50403421 | 8.41491045 |  |  |  |  |
|  |  | 10:50403564 | 7.05736921 |  |  |  |  |
|  |  | 10:50413514 | 7.15455321 |  |  |  |  |
|  |  | 10:50421561 | 7.02001578 |  |  |  |  |
|  |  | 10:50425279 | 7.7456717 |  |  |  |  |
|  |  | 10:50425285 | 7.59734295 |  |  |  |  |
